# Homeostasis model assessment to detect insulin resistance and identify patients at high risk of breast cancer development: National Cancer Institute of Naples experience

**DOI:** 10.1186/1756-9966-32-14

**Published:** 2013-03-14

**Authors:** Immacolata Capasso, Emanuela Esposito, Francesca Pentimalli, Maurizio Montella, Anna Crispo, Nicola Maurea, Massimiliano D’Aiuto, Alfredo Fucito, Maria Grimaldi, Ernesta Cavalcanti, Giuseppe Esposito, Giuseppe Brillante, Sergio Lodato, Tonino Pedicini, Giuseppe D’Aiuto, Gennaro Ciliberto, Antonio Giordano

**Affiliations:** 1 Department of Senology, National Cancer Institute, ‘Pascale Foundation’, Via Mariano Semmola, Naples, 80131, Italy; 2 INT-CROM, National Cancer Institute, ‘Pascale Foundation’, Cancer Research Center, Via Ammiraglio Bianco, Mercogliano, Avellino, 83013, Italy; 3 Department of Epidemiology, National Cancer Institute, ‘Pascale Foundation’, Naples, Italy; 4 Department of Cardiology, National Cancer Institute, ‘Pascale Foundation’, Naples, Italy; 5 Department of Clinical Pathology, National Cancer Institute, ‘Pascale Foundation’, Naples, Italy; 6 General Director,National Cancer Institute, ‘Pascale Foundation’, Naples, Italy; 7 Scientific Director; National Cancer Institute, ‘Pascale Foundation’, Naples, Italy; 8 Sbarro Institute for Cancer Research and Molecular Medicine, College of Science and Technology, Temple University, BioLife Science Bldg. Suite 333 1900 N 12th Street, Philadelphia, PA, USA; 9 Department of Human Pathology and Oncology, University of Siena, Siena, Italy

**Keywords:** Metabolic syndrome, Insulin resistance, Breast cancer, Postmenopausal, HOMA-IR

## Abstract

**Background:**

Metabolic Syndrome (MS) has been correlated to breast carcinogenesis. MS is common in the general population (34%) and increases with age and body mass index. Although the link between obesity, MS and hormone related cancer incidence is now widely recognized, the molecular mechanisms at the basis of such increase are still poorly characterized. A crucial role is supposed to be played by the altered insulin signalling, occurring in obese patients, which fuels cancer cell growth, proliferation and survival. Therefore we focused specifically on insulin resistance to investigate clinically the potential role of insulin in breast carcinogenesis.

**Methods:**

975 patients were enrolled and the association between MS, insulin resistance, and breast cancer was evaluated. Women were stratified by age and menopausal status. Insulin resistance was measured through the Homeostasis Model Assessment score (HOMA-IR). The cut off value to define insulin resistance was HOMA-IR ≥ 2.50.

**Results:**

Higher prevalence of MS (35%) was found among postmenopausal women with breast cancer compared to postmenopausal healthy women (19%) [OR 2.16]. A broad range of BMI spanning 19–48 Kg/m^2^ was calculated. Both cases and controls were characterized by BMI ≥ 25 Kg/m^2^ (58% of cases compared to 61% of controls). Waist circumference >88 cm was measured in 53% of cases - OR 1.58- (95% CI 0.8-2.8) and in 46% of controls. Hyperinsulinemia was detected in 7% of cases – OR 2.14 (95% CI 1.78-2.99) and only in 3% of controls. HOMA-IR score was elevated in 49% of cases compared to 34% of controls [OR 1.86], suggesting that insulin resistance can nearly double the risk of breast cancer development. Interestingly 61% of women operated for breast cancer (cases) with HOMA-IR ≥ 2.5 presented subclinical insulin resistance with fasting plasma glucose levels and fasting plasma insulin levels in the normal range. Both android fat distribution and insulin resistance correlated to MS in the subgroup of postmenopausal women affected by breast cancer.

**Conclusions:**

Our results further support the hypothesis that MS, in particular insulin resistance and abdominal fat, can be considered as risk factors for developing breast cancer after menopause. We suggest that HOMA-IR, rather than fasting plasma glucose and fasting plasma insulin levels alone, could be a valuable tool to identify patients with subclinical insulin resistance, which could be relevant for primary prevention and for high risk patient screening.

## Background

Metabolic syndrome (MS), characterized by central adiposity, insulin resistance, low serum high density lipoprotein cholesterol (HDL-C), high serum triglycerides, and high blood pressure, seems to be in strict correlation to breast carcinogenesis [1,2]. MS, according to the National Cholesterol Program (NCEP) Adult Treatment Panel III (ATP III), can be defined as the presence of at least three of the following clinical criteria: waist circumference >88 cm in women, HDL-C <50 mg/dl, blood pressure ≥130/85 mmHg, triglyceride >150 mg/dl and insulin resistance [3]. The prevalence of MS is high in the general population with approximately 34% of adults meeting the above-mentioned criteria and increases with age and body mass index (BMI). In fact, women over 60 years and overweight or obese are much more likely to meet the MS criteria [4]. Consistently, post-menopausal women are often affected by MS and, interestingly, show the highest incidence of breast cancer in the female population [1]. Although many epidemiological studies link obesity and MS to the increased frequency of many cancer types, the molecular mechanisms underlying this increased risk are still poorly characterized. Visceral adipose tissue has multiple endocrine, metabolic and immunological functions and has been shown to be central in the MS pathogenesis. MS is a pro-inflammatory, pro-coagulant state associated with insulin resistance [5,6]. The increase in adipose tissue mass, which characterizes MS, can have both direct and secondary effects favouring tumorigenesis [6]. Obese patients often develop insulin resistance with various tissues showing low cell sensitivity to insulin activity. As a consequence, a balancing mechanism stimulates insulin release resulting in a chronic compensatory hyperinsulinemia. By continuously stimulating insulin signalling in sensitive tissues, high levels of circulating insulin cause aberrantly increased mitogenic and antiapoptotic effects [7]. Although the obese state generates peripheral insulin resistance in many tissues, not all insulin signalling is impaired. In the diabetic liver, the gluconeogenic pathway becomes insulin resistant, and insulin-stimulated lipogenesis remains sensitive. Thus, in insulin-resistant patients, specific tissues and signalling pathways can remain insulin-sensitive and are exposed to higher than normal levels of insulin signalling. Initial experiments demonstrated that in human breast cancer cell lines insulin has been shown to promote DNA synthesis, suggesting a mitogenic effect [6]. When insulin concentrations are high, insulin — which is structurally similar to insulin-like growth factor 1 and 2 (IGF1 and IGF2) — acts also as a growth factor by binding the IGF-receptors (IGF1R and IGF2R) [8,9]. Moreover, increased insulin signalling can induce overexpression of the receptors [9]. Consistently, in vitro and in vivo studies have shown insulin receptor overexpression in breast tissue. Furthermore, it seems that high insulin levels can alter the levels of IGF-binding proteins, which regulate the amount of bioactive insulin or IGFs in the microenvironment, thereby resulting in impaired insulin signalling [6]. As various epidemiological studies associated type 2 diabetes with increased incidence of various cancer types, including breast cancer, we wondered what is the specific contribution of insulin resistance in breast carcinogenesis at the clinical level [10-12]. To this aim we compared breast cancer patients to healthy women in order to assess whether a correlation exist with MS criteria and, specifically, insulin resistance measured through Homeostasis Model Assessment (HOMA-IR).

## Methods

### Enrollment and exclusion criteria

975 women spanning 35–75 years in age have been enrolled in our nested case–control observational retrospective study between 2008 and 2011 (Table 1). 410 women underwent surgery for breast cancer (cases), whereas 565 were healthy women (controls). Healthy women referred to National Cancer Institute for the breast cancer screening program. Women aged over forty had clinical examination, mammogram and ultrasound. Women under forty had clinical examination and ultrasound. Cases were patients with histological diagnosis of breast cancer at age of recruitment. Controls were patients with completely negative clinical-instrumental reports and no familial history of breast or ovarian cancer. None of the controls has developed a breast cancer till today. In accordance with the Helsinki Declaration of 1975, after obtaining informed consent, for each woman anthropometric features were measured, including weight in kilograms, height in meters, waist and hip circumference; arterial blood pressure was taken and venous blood was collected on study entry. Body Mass Index (BMI) (kg/m^2^) was calculated from weight and height values and evaluated according to the World Health Organization classification (<25 kg/m^2^ = underweight/normal, ≥25 kg/m^2^ = overweight/obese). The waist and hip ratio (WHR) was obtained from waist and hip circumference, measuring the smallest circumference of both to discriminate between android and gynoid fat distribution. Fasting plasma glucose, insulin levels, HDL-C, triglycerides, were assessed from blood samples. In particular, fasting plasma glucose, HDL-C and triglycerides were measured according to the NCEP ATP III criteria. Blood samples were locally assessed at the central laboratory of the National Cancer Institute. Sample collection was standardized by time at blood withdrawing. Samples were taken in the early morning hours (between 8.00 and 10.00 A.M.). Fasting plasma glucose assessment was measured by the COBAS INTEGRA Glucose HK cassette (GLUC2). It contains an in vitro diagnostic reagent system intended for use on COBAS INTEGRA systems for the quantitative determination of the glucose concentration in hemolysate. Electrochemiluminescence immunoassay (ECLIA) applied on Cobas 6000 was used for insulin concentration measurement. Enzymatic colorimetric test CHOD – POD was employed for cholesterol dosage. The GPO - POD method based on the enzymatic determination of glycerol using the enzyme glycerol phosphate oxidase (GPO) was used for triglycerides determination. Fresh, clear, unhemolyzed serum was the specimen of choice. The specimen was collected following the guidelines of NCCLS document H4-A3. Diabetes was considered an exclusion criterion. Diabetes was diagnosed on laboratory determinations with fasting plasma glucose assessment ≥ 126 mg/dl according to American Diabetes Association guidelines [13]. Fasting plasma glucose levels in the range between 110 and 126 mg/dl were considered as hyperglycaemia. Insulin levels were defined in the normal range when between 5 and 25 mcU/ml, whereas concentrations above 25 mcU/ml were considered corresponding to hyperinsulinemia.

**Table 1:** Age at recruitment and menopausal status by cases and controls

**Menopausal status**				
	**Controls**		**Cases**	
	n.	%	n.	%
PRE	229	40.5	124	30.2
POST	336	59.5	286	59.8
Total	565	100	410	100
				
**Age at recruitment**				
	**Controls**		**Cases**	
	**n.**	**%**	**n.**	**%**
**< 35**	**18**	**3.2**	**13**	**3.2**
**35-44**	**104**	**18.4**	**70**	**17.1**
**45-54**	**217**	**38.4**	**99**	**24.1**
**55-64**	**166**	**29.4**	**100**	**24.4**
**≥65**	**60**	**10.6**	**128**	**31.2**
**Total**	**565**	**100**	**410**	**100**

### HOMA – IR and statistical analysis

After data collection, we used the HOMA-IR, Homeostasis Model Assessment of insulin resistance, to quantify insulin resistance [14]. The HOMA-IR score was calculated as the product of the fasting plasma insulin level (mcU/mL) and the fasting plasma glucose level (mg/dl), divided by 405. The cut off value to define insulin resistance was HOMA-IR ≥ 2.50. Patients presenting HOMA-IR ≥ 2.50 were considered insulin resistant. Chi-squared test and logistic regression analyses (OR and 95% CI) were used to confirm the association between MS and breast cancer and to calculate the risk. Regression analyses were adjusted for age, menopausal status and BMI. Statistical significance was considered at p < 0.05.

## Results

565 healthy women and 410 patients affected by breast cancer were enrolled between 2008 and 2011 in our nested case–control observational retrospective study. Our first end point consisted in updating our previous results about the association between MS and breast cancer. Second end point was focusing on insulin resistance that is the most important feature characterizing MS relation to cancer. Among the 975 women included in the study 286 cases and 336 controls were defined as menopausal (mean age 57.6 years) with Odds Ratio of postmenopausal breast cancer of 1.63 (95% CI 1.09- 1.79). Overall, considering the 975 women included in the study (age range = 35–75 years) MS prevalence was higher among cases (27%) than in controls (14%). We did not find significant differences in MS prevalence between cases and controls among premenopausal patients, whereas the prevalence of MS in postmenopausal was 35% for cases OR 2.16 (95% CI = 0.31 to 0.39) and 19% for controls (95% CI = 0.16 to 0.23). MS was detected in one third of post-menopausal cases. A broad range of BMI spanning 19–48 Kg/m^2^ was calculated. Both cases and controls were characterized by high BMI (58% of cases compared to 61% of controls). Waist circumference >88 cm was measured in 53% of cases - OR 1.58- (95% CI 0.8-2.8) and in 46% of controls. Hypertriglyceridemia was found in 14% of cases respect to 9% of controls [OR 1.4]. 27% of cases presented HDL-C <50 mg/dl compared to 24% of controls [OR 1.09]. High blood pressure was detected in 40% of cases – OR 1.58 (95% CI 0.37-0.47) respect to 30% of controls. Hyperinsulinemia was detected in 7% of cases – OR 2.14 (95% CI 1.78-2.99) and only in 3% of controls (Table 2).

**Table 2:** Metabolic variables by case–control status

	**Cases (410)**		**Controls (565)**		
	**N°**	**%**	**N°**	**%**	**p-value**
**Fasting plasma glucose (mg/dl)**					
**< 110**	**345**	**84.1**	**508**	**90.0**	
**≥ 110**	**65**	**15.9**	**57**	**10.0**	**<0.001**
**Insulin**					
**0-25 regular**	**386**	**94.2**	**545**	**96.5**	
**≥ 25 hyperinsulinemia**	**24**	**5.8**	**20**	**3.5**	**0.13**
**High blood pressure**					
**Yes**	**161**	**39.4**	**180**	**31.8**	**0.01**
**No**	**249**	**60.6**	**385**	**68.2**	
**Tryglicerides**					
					
**≤150**	**354**	**86.4**	**508**	**90.4**	
**>150**	**56**	**13.6**	**57**	**9.6**	**0.006**
**HDL-Col**					
**< 50 mg/dL**	**109**	**26.5**	**140**	**24.9**	
**≥ 50 mg/dl**	**301**	**73.5**	**425**	**75.1**	**0.9**
**WC**					
**≤ 88 cm**	**195**	**47.7**	**304**	**53.8**	**0.003**
**>88 cm**	**215**	**52.3**	**261**	**46.2**	
**BMI**					
**≤ 25**	**172**	**42.0**	**222**	**39.3**	**0.7**
**>25**	**238**	**58.0**	**343**	**60.7**	
**WHR**					
**<0.8**	**99**	**24.2**	**118**	**20.9**	
**≥0.8**	**311**	**75.8**	**447**	**79.1**	**0.001**
**Metabolic syndrome criteria**					
**0-2**	**301**	**73.4**	**484**	**85.70**	
**3-5**	**109**	**26.6**	**81**	**14.3**	**< 0.001**

HDL-Chol = HDL-Cholesterol; BMI = Body Mass Index; WC = Waist Circumference; WHR = Waist Hip Ratio.

**Figure 1:**
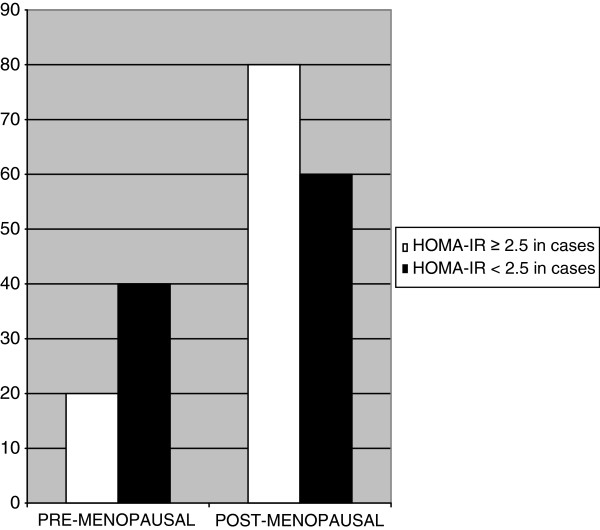
HOMA- IR as indicator of insulin resistance in pre and post-menopausal patients with breast cancer.

HOMA-IR was ≥ 2.50 in 49% of cases – OR 1.86 (C.I.95% =0.42 to 0.52) respect to 34% of controls (C.I.95% =0.03 to 0.38), showing a positive trend for breast cancer patients. Interestingly, 80% of insulin resistant cases were postmenopausal, whereas premenopausal were only 20% (C.I.95% =0.85 to 0.74 vs 0.33 to 0.7) (Figure 1).

**Figure 2:**
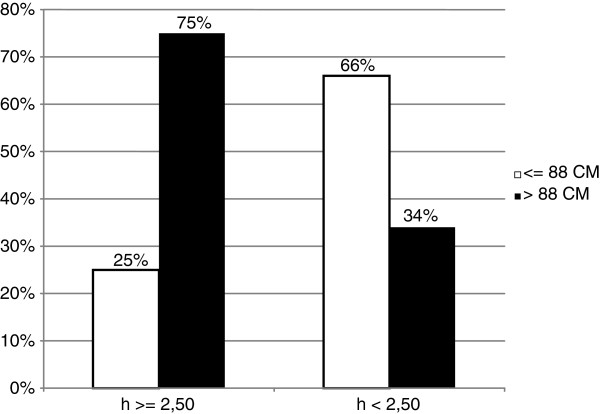
**Histogram comparing insulin resistance and waist circumference among breast cancer patients.** Statistical significance (P < 0.05) for comparison waist circumference in insulin resistant patients.

HOMA-IR and insulin were positively associated to at least three other MS criteria in 89% of cases compared to 50% of controls. Remarkably, 75% of cases were insulin resistant (HOMA-IR ≥ 2.5) with waist circumference > 88 cm (Table 3, Figure 2).

**Table 3:** HOMA-IR by categories of waist circumference

	**WAIST CIRCUMFERENCE**		
**HOMA-IR**	**≤ 88cm**	**>88cm**	Total
**≥ 2.50**	51 (25%)	150 (75%)	201
**< 2.50**	137 (66%)	72 (34%)	209
Total	188	222	

Insulin resistant cases and controls have been further stratified in four subgroups according to fasting plasma glucose and insulin values. In particular, group 1 had fasting plasma glucose levels and fasting plasma insulin levels in the normal range; group 2 had high levels of both fasting plasma glucose and fasting plasma insulin; group 3 had fasting plasma glucose in the normal range and high levels of fasting plasma insulin; group 4 had fasting plasma insulin in the normal range and high levels of fasting plasma glucose. Interestingly, 61% of women operated for breast cancer (cases) with HOMA-IR ≥ 2.5 presented fasting plasma glucose levels and fasting plasma insulin levels in the normal range (group 1). Only 5% of cases showed high levels of both fasting plasma glucose and fasting plasma insulin (group 2). 7% were euglycemic, but plasmatic insulin levels were high (group 3). 27% of patients presented as hyperglycaemic, but insulin levels were in the normal range (group 4).

## Discussion

Our data still confirm the existing linkage between metabolic alterations and breast cancer. Higher prevalence of MS (35%) among postmenopausal women with breast cancer compared to healthy women (19%) [OR 2.16] was found. No statistical significant difference in premenopausal women was found. Probably, alterations in metabolic signalling that activate pro-mitotic and anti-apoptotic pathways are more likely to occur in postmenopausal women. Moreover all MS features were positively, but weakly associated to breast cancer risk. As expected from the recent literature, android fat distribution-consisting in WC >88 cm- was positively associated to MS and breast cancer more than BMI. Waist circumference >88 cm was measured in 53% of cases - OR 1.58 (95% CI 0.8-2.8) and in 46% of controls, whereas no differences in BMI were found between cases and controls. A majority of prospective studies show breast cancer risk to be higher in obese postmenopausal women with upper abdominal adiposity than in those with overall adiposity. The evidence is more limited and inconsistent in the case of premenopausal women. Overall adiposity in women adversely affects breast cancer risk mainly by greater exposure of mammary epithelial tissue to endogenous oestrogen and to pro-inflammatory cytokines. Upper abdominal adiposity appears to involve an additional effect related to the presence of insulin resistance [15]. Waist circumference measurement reveals to be more accurate than BMI alone in breast cancer risk evaluation. Second end point of our study was to singularly analyze insulin resistance contribution in determining breast cancer risk. 49% of cases were insulin resistant respect to 34% of controls [OR 1.86], suggesting that insulin resistance can nearly double the risk of breast cancer development. 80% of the insulin resistant patients were postmenopausal, but the most important aspect to consider is that 61% of women operated for breast cancer (cases) with HOMA-IR ≥ 2.5, and so to be considered as insulin resistant, presented fasting plasma glucose levels and fasting plasma insulin levels in the normal range, whereas 7% of patients were euglycemic, but plasmatic insulin levels were high. Consequently 68% of patients had levels of fasting plasma glucose in the normal range, and, similarly, fasting plasma insulin levels were diagnosed as normal in 88% of cases. Only through the use of HOMA score were we able to diagnose insulin resistance. These patients were defined insulin resistant only by using HOMA-IR score. That means subclinical insulin resistance can be misunderstood. Last published data by our research group in 2010 did not consider single features of MS per se in correlation to breast cancer [1]. Now we have focused on the association between insulin resistance and breast cancer and a positive correlation between insulin resistance and breast cancer patients was found. Both android distribution fat and insulin resistance correlated to MS in the subgroup of postmenopausal women affected by breast cancer and were positively and independently associated with more than three other MS criteria [3,16,17].

## Conclusions

Our data, consistently with our previous study, further support the hypothesis that MS can be considered as a risk factor for developing breast cancer in postmenopause [1]. We specifically focused on the insulin resistance phenotype, the condition of chronic hyperinsulinemia to which cells are exposed in response to low cell sensitivity to insulin activity [18,19]. Insulin resistance can often be defined as a subclinical condition. Consistently, most of our patients (68%) had levels of fasting plasma glucose in the normal range, and, interestingly, only through the use of HOMA score we classified them as insulin resistant. Similarly, fasting plasma insulin levels were diagnosed as normal in 88% of cases. These patients were identified as insulin resistant only by means of the HOMA score. HOMA-IR is widely-used in epidemiologic studies as a measure of insulin resistance, and has been shown to reflect euglycemic clamp insulin resistance more accurately than fasting insulin levels alone. In conclusion, our experience suggests that insulin resistance and abdominal fat (more than BMI alone) represent the most important criteria of MS on which primary prevention should be concentrated. Interestingly, Homeostasis Model Assessment of insulin resistance promises to be a valuable tool for primary prevention, particularly for patients with subclinical insulin resistance, presenting fasting plasma glucose levels and fasting plasma insulin levels in the normal range. Our findings suggest that HOMA-IR could be useful in screening patients at higher risk of developing breast cancer.

## Abbreviations

HOMA-IR: Homeostasis model assessment – insulin resistance; NCEP: National cholesterol program; ATP III: Adult treatment panel III; IGF1: Insulin-like growth factor 1; IGF2: Insulin-like growth factor 2; MS: Metabolic syndrome; BMI: Body mass index; WC: Waist circumference; WHR: Waist hip ratio; LDL cholesterol: Low density lipoprotein - cholesterol; HDL cholesterol: High density

## Competing interests

The authors declare that they have no competing interests.

## Authors’ contributions

IC realized the protocol design, EE wrote the draft and edited the manuscript. FP revised critically the manuscript. AG has given final approval of the version to be published. MM, AC and MG contributed to the statistical design. NM recruited metabolic syndrome affected women. GDA and GC conceived the study idea, supervised the study design. SL and TP supervised the protocol development. MDA and AF recruited patients for the study and selected patients at risk of breast cancer. EC and GE took blood samples and analyzed them in the lab. GB has contributed in data managing and preparing informed consent. All authors read and approved the final manuscript.
